# How to Reduce the Transmission Risk of COVID-19 More Effectively in New York City: An Age-Structured Model Study

**DOI:** 10.3389/fmed.2021.641205

**Published:** 2021-08-13

**Authors:** Miaolei Li, Jian Zu, Zongfang Li, Mingwang Shen, Yan Li, Fanpu Ji

**Affiliations:** ^1^School of Mathematics and Statistics, Xi'an Jiaotong University, Xi'an, China; ^2^National & Local Joint Engineering Research Center of Biodiagnosis and Biotherapy, The Second Affiliated Hospital of Xi'an Jiaotong University, Xi'an, China; ^3^Key Laboratory of Environment and Genes Related to Diseases, Ministry of Education of China, Xi'an Jiaotong University, Xi'an, China; ^4^School of Public Health, Health Science Center, Xi'an Jiaotong University, Xi'an, China; ^5^Department of Population Health Science and Policy, Icahn School of Medicine at Mount Sinai, New York, NY, United States; ^6^Department of Obstetrics, Gynecology, and Reproductive Science, Icahn School of Medicine at Mount Sinai, New York, NY, United States; ^7^Department of Infectious Diseases, The Second Affiliated Hospital of Xi'an Jiaotong University, Xi'an, China

**Keywords:** COVID-19, age-structured model, releasing strategies, social distancing, mobile cabin hospital, second wave

## Abstract

**Background:** In face of the continuing worldwide COVID-19 epidemic, how to reduce the transmission risk of COVID-19 more effectively is still a major public health challenge that needs to be addressed urgently.

**Objective:** This study aimed to develop an age-structured compartment model to evaluate the impact of all diagnosed and all hospitalized on the epidemic trend of COVID-19, and explore innovative and effective releasing strategies for different age groups to prevent the second wave of COVID-19.

**Methods:** Based on three types of COVID-19 data in New York City (NYC), we calibrated the model and estimated the unknown parameters using the Markov Chain Monte Carlo (MCMC) method.

**Results:** Compared with the current practice in NYC, we estimated that if all infected people were diagnosed from March 26, April 5 to April 15, 2020, respectively, then the number of new infections on April 22 was reduced by 98.02, 93.88, and 74.08%. If all confirmed cases were hospitalized from March 26, April 5, and April 15, 2020, respectively, then as of June 7, 2020, the total number of deaths in NYC was reduced by 67.24, 63.43, and 51.79%. When only the 0–17 age group in NYC was released from June 8, if the contact rate in this age group remained below 61% of the pre-pandemic level, then a second wave of COVID-19 could be prevented in NYC. When both the 0–17 and 18–44 age groups in NYC were released from June 8, if the contact rates in these two age groups maintained below 36% of the pre-pandemic level, then a second wave of COVID-19 could be prevented in NYC.

**Conclusions:** If all infected people were diagnosed in time, the daily number of new infections could be significantly reduced in NYC. If all confirmed cases were hospitalized in time, the total number of deaths could be significantly reduced in NYC. Keeping a social distance and relaxing lockdown restrictions for people between the ages of 0 and 44 could not lead to a second wave of COVID-19 in NYC.

## Introduction

The 2019 novel coronavirus disease (COVID-19) is an emergent and virulent infectious disease caused by SARS-CoV-2. Since its outbreak in January 2020, it has rapidly spread to more than 100 countries and regions (1–3). New York City (NYC) was the epicenter of the COVID-19 pandemic in the United States. In early April 2020, the daily confirmed cases in NYC rose above 6,000, daily deaths in NYC reached more than 500, which resulted in huge challenges to public health security and limited health care resources (4). Although NYC has implemented a series of prevention and control measures, such as the stay-at-home order and mask mandate in public settings (5, 6), it was still not enough to effectively control the spread of COVID-19.

During the COVID-19 outbreak in Wuhan, China, the Chinese government had assembled several medical teams, quickly established several mobile cabin hospitals, and conducted centralized isolation and scientific treatment for confirmed mild cases. Several studies have shown that mobile cabin hospitals played an important role in controlling China's outbreak of COVID-19 infection under the policy of ensuring that all infected people are diagnosed, isolated, hospitalized or treated (7–9). A retrospective study among 483 patients with COVID-19 from the mobile cabin hospital in Wuhan, Wang et al. showed that the mobile cabin hospital could effectively treat and isolate these patients, as well as reduce severe cases and mortality (7). Sun et al. summarized the experience of mobile cabin hospitals in Wuhan and showed that mobile cabin hospitals had effectively alleviated the shortage of medical resources and allowed for a centralized management of confirmed mild cases (8). Wang et al. reviewed the medical records of 421 patients with COVID-19 admitted to a mobile cabin hospital in Wuhan, they showed that mobile cabin hospitals could effectively treat patients with COVID-19 who had mild symptoms and prevented the spread of the SARS-CoV-2 (9). However, the impact of all diagnosed and all hospitalized on the transmission risk of COVID-19 in NYC remained unclear.

Moreover, a second wave greater than the current practice has occurred in the United States currently, which suggested that the current relaxing lockdown restriction strategy could be improved. Limited research focused on the release policies by age for COVID-19 control (10–13), so we wanted to further assess how to minimize the risk of a second wave of COVID-19 in NYC when different age groups were released on June 8th, 2020.

In general, this study aimed to explore how to reduce the transmission risk of COVID-19 more effectively in NYC. Specifically, we developed an age-structured model and assessed the impact of all diagnosed and all hospitalized on the transmission risk of COVID-19 in NYC. Moreover, we evaluated the impact of reopening the economy for different age groups on the risk of a second wave of COVID-19 in NYC. The results of this study will provide a quantitative reference for government agencies in NYC as well as in other countries and regions to reduce the transmission risk of COVID-19.

## Methods

### Reported Data

The reported data for COVID-19 used in this study were collected from the official website of the City of New York (4, 14), including the cumulative number of confirmed cases (Supplementary Data.xlsx, columns 2–6), the cumulative number of deaths (Supplementary Data.xlsx, columns 7–11) and the cumulative number of hospitalizations (Supplementary Data.xlsx, columns 12–16) for 5 age groups (0–17, 18–44, 45–64, 65–74, and 75–100) from March 24, 2020 to June 7, 2020. From the reported data we could see that the age group over 65 had a higher mortality rate, the total number of confirmed cases was higher between the ages of 18 and 64 (see Supplementary Figure 1) (10, 15–17). All of these data were used to estimate the unknown parameters and initial values of the mathematical model.

### Model Structure and Assumptions

**Figure 1:**
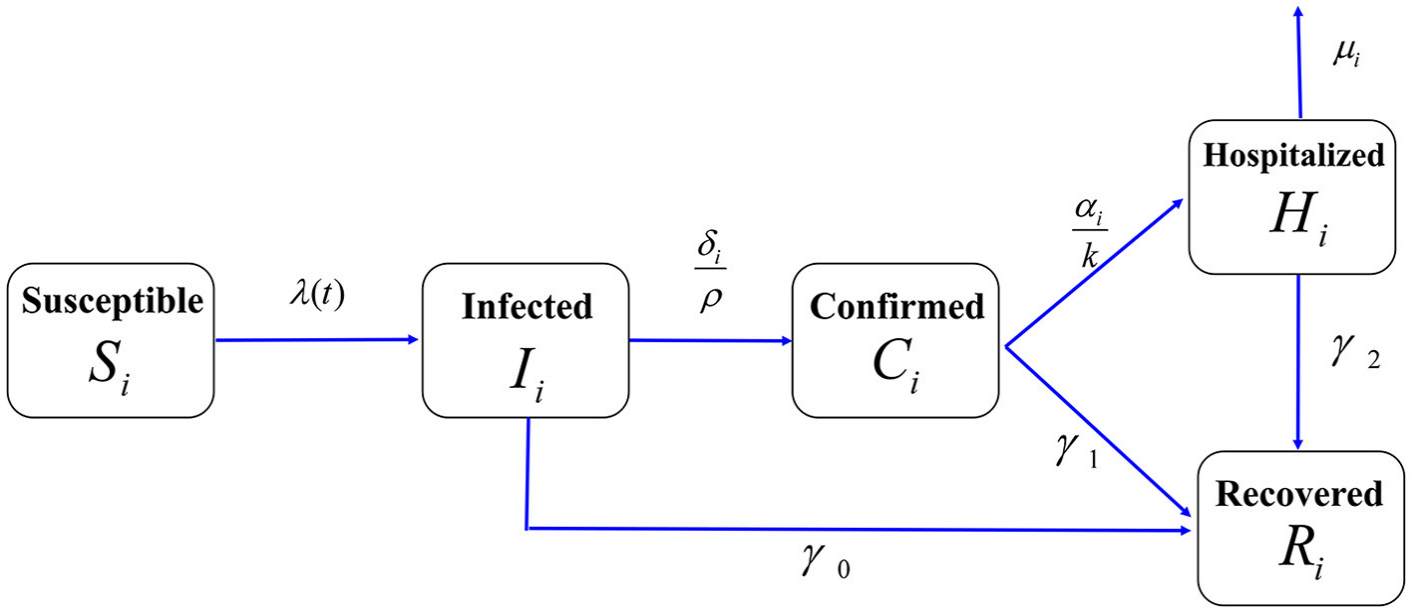
Flow chart of the age-structured COVID-19 transmission model in NYC. The total population was divided into five compartments: Susceptible individuals (*S*), Infected individuals (*I*), Confirmed cases but stayed at home (*C*), Hospitalized cases (*H*), and Recovered cases (*R*). The population of each compartment was further divided into five age groups. We assumed that the confirmed cases that stay at home can cause household infection. The contact rate (*c*_*ij*_), death rate (μ_*i*_*)*, diagnosis rate (δ_*i*_) and hospitalization rate (α_*i*_) were assumed to be age-related.

Based on the transmission characteristics of COVID-19 and age-specific reported data in NYC, we developed an age-structured susceptible-infected-confirmed-hospitalized-recovered (SICHR) model at the population level (a detailed model description was provided in the first and second section of Supplementary Material) (18–26). The model structure for each age group was depicted in Figure 1. Based on the COVID-19 reported data from March 24 to June 7, 2020 in NYC (Supplementary Data), we used the Markov Chain Monte Carlo (MCMC) approach to estimate the unknown parameter values and initial values of the SICHR model as well as their 95% confidence intervals (Supplementary Table 1). The detailed description of other variables and their sources were summarized in the third section of Supplementary Material.

### Model Calibration

We considered the COVID-19 outbreak in NYC from March 24 to June 7 (before the first releasing) as the baseline for this study. We compared the estimated values with the three types of reported data for COVID-19 in NYC, including the cumulative number of confirmed cases (Supplementary Figure 2), the cumulative number of deaths and the cumulative number of hospitalizations for five age groups from March 24, 2020 to June 7, 2020 (Supplementary Figures 3, 4). The results showed that the estimated values fitted the reported data very well. Therefore, the mathematical model and estimated parameters were credible and can be used to explore how to reduce the transmission risk of COVID-19 more effectively.

### Impact of All Diagnosed and All Hospitalized

We considered two scenarios: one was that all confirmed cases could be hospitalized from three different times, that was, from March 26, April 5 and April 15, 2020, respectively; the other was that all infected people were confirmed from March 26, April 5 and April 15, 2020, respectively. We chose March 26, April 5, and April 15 because these dates were within a few days before and after the peak of the number of new confirmed cases in NYC. Besides, confirmed cases with mild symptoms were considered to be staying at home due to limited medical resources during the pandemic. However, this may lead to potential secondary household infections. Moreover, several studies have shown that mobile cabin hospitals could effectively alleviated the shortage of medical resources and conducted centralized isolation and scientific treatment for confirmed mild cases, which could effectively treat patients with COVID-19 who had mild symptoms (7–9). Therefore, we assumed that if NYC imitated the experience from the Wuhan mobile cabin hospitals, and there were enough mobile cabin hospitals conducting centralized isolation and scientific treatment for confirmed mild cases in NYC, just like in Wuhan, China, then the spread of the COVID-19 would have been prevented effectively.

To evaluate the impact of all hospitalized, we estimated the cumulative number of deaths for five age groups by assuming that the death rate of hospitalized cases was a decreasing function over time when more healthcare resources became available (27–29). Here, we assumed that the healthcare resources in NYC were adequate from March 26, April 5, and April 15, 2020, respectively. Particularly, we used an exponentially decreasing function μ_*i*_(*t*) = μ_*i*_exp(−*at*)+*b* to describe the mortality in hospitalized cases, where *a* was the exponential decline rate and *b* was the minimal death rate due to infection. By fitting the case fatality ratio (reported deaths among total cases) for COVID-19 in Wuhan, China (29), we obtained *a* = 0.0665 (95%CI, 0.0633–0.0697) and *b* = 0.00020 (95% CI, 0.00017–0.00023). Besides, when the confirmed cases who stayed at home were quarantined and treated at hospital, we no longer considered their deaths. To evaluate the impact of all diagnosed, we also estimated the daily number of new infections for five age groups by assuming that all infected people were confirmed from March 26, April 5, and April 15, 2020, respectively.

### Impact of Release of Different Age Groups

In order to evaluate the risk of a second wave of COVID-19 in NYC when different age groups were released from June 8, 2020, we estimated the daily number of new confirmed cases for different age groups. Due to the higher mortality rate in the age group over 65 years old, here we no longer considered the case of releasing the age group over 65 years old separately. Specifically, we considered three scenarios: releasing 0–64 age groups, releasing only one age group, releasing two age groups. In addition, we assumed that the contact rates had been reduced by 80% during the time of COVID-19 epidemic (30–32). In particular, compared with the current practice, we estimated that under what level of contact rate, a smaller second wave or a larger second wave of COVID-19 would have been prevented in NYC.

### Sensitivity Analysis

Considering the healthcare capacity of NYC hospitals, we further performed sensitivity analysis to evaluate the cumulative number of deaths in NYC by assuming that 100, 90, 80, and 70% of all confirmed cases were hospitalized from March 26, April 5, and April 15, 2020, respectively. Here, based on the exponentially decreasing death rate μ_*i*_(*t*) = μ_*i*_exp(−*at*)+*b*, we assumed that the exponential decline rate of death rate was 100 *% a*, 90 *% a*, 80 *% a* and 70 *% a*, respectively.

## Results

### Impact of All Hospitalized

**Figure 2:**
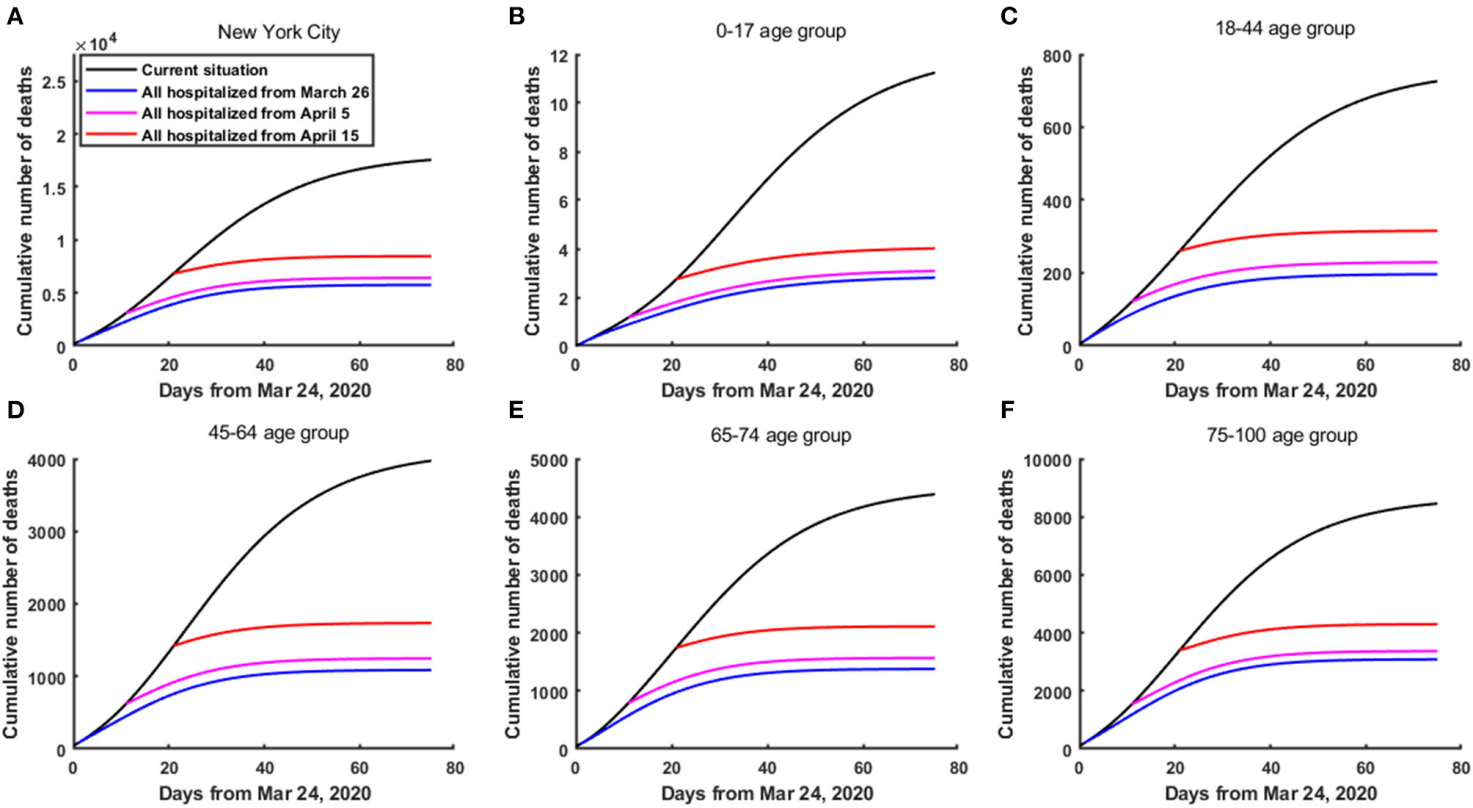
The cumulative number of deaths in NYC if all confirmed cases were hospitalized from March 26, April 5 and April 15, 2020, respectively. **(A)** In the whole population of NYC. **(B)** In the 0–17 age group. **(C)** In the 18–44 age group. **(D)** In the 45–64 age group. **(E)** In the 65–74 age group. **(F)** In the 75–100 age group.

The results showed that in comparison with the current practice, if all confirmed cases were hospitalized from March 26, April 5, and April 15, 2020; then as of June 7, the total number of deaths in NYC decreased by 11,802, 11,134, and 9,091, respectively, and the corresponding percentages reduction were 67.24, 63.43, and 51.79%, respectively (Figure 2A). Particularly, for the 75–100 age group, compared with the current practice, the total number of deaths in NYC reduced by 5,367, 5,083, and 4,158, respectively (Figure 2F). Correspondingly, the percentages reduction were 63.48, 60.13, and 49.18%. It could be seen that the earlier the hospitalization of all confirmed cases, the greater the reduction in the total number of deaths (Figures 2A–F).

### Impact of All Diagnosed

**Figure 3:**
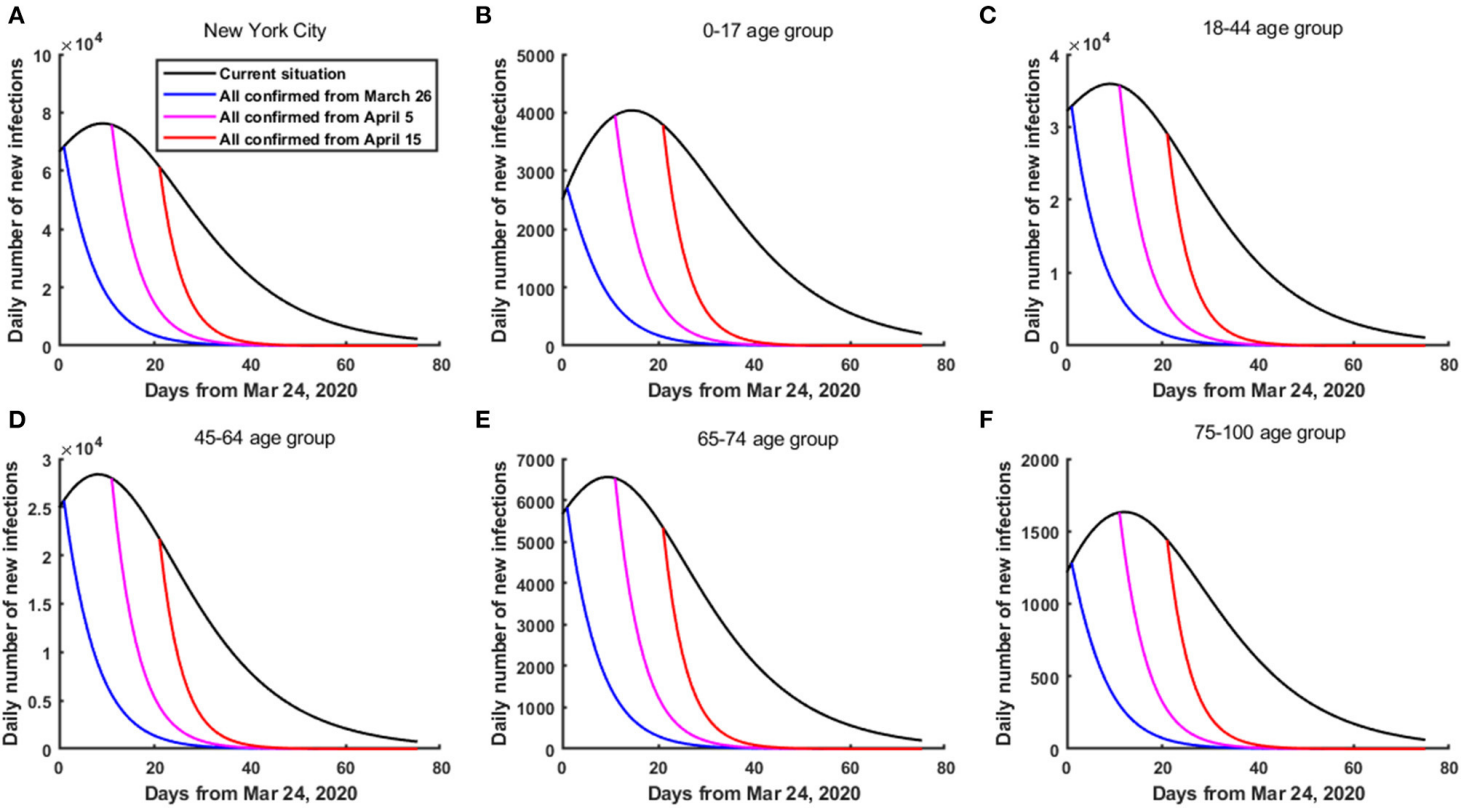
The daily number of new infections in NYC if all infected people were diagnosed from March 26, April 5 and April 15, 2020, respectively. **(A)** In the whole population of NYC. **(B)** In the 0–17 age group. **(C)** In the 18–44 age group. **(D)** In the 45–64 age group. **(E)** In the 65–74 age group. **(F)** In the 75–100 age group.

The results showed that under the current practice, on April 22, 2020, the number of new infections in NYC reached 43,795. If all infected people were diagnosed from March 26, April 5, and April 15, 2020, then the number of new infections on April 22 in NYC were only 868, 2,679 and 11,353, respectively, a decrease of 98.02, 93.88, and 74.08%, respectively. In addition, as of June 7, 2020, the cumulative number of infected individuals in NYC reduced by 2,110,764, 1,761,081, and 1,508,130, respectively, a decrease of 79.37, 66.22, and 56.71% (Figure 3A). Particularly, for the 18–44 age group, compared with the current practice, if all infected people were diagnosed from March 26, April 5, and April 15, 2020, then on April 22, 2020, the number of new infections in 18–44 age group reduced by 20,451, 19,593, and 15,465, a decrease of 98.05, 93.94, and 74.15%, respectively. Moreover, as of June 7, 2020, the cumulative number of infected individuals in 18–44 age group reduced by 1,002,674, 837,534, and 717,315, respectively, a decrease of 79.40, 66.32, and 56.80% (Figure 3C). We can see that the earlier the diagnosis of all infected people, the greater the reduction in the total number of infected individuals (Figures 3A–F). However, it should be reemphasized the fact that for the purpose of this analysis, confirmed cases were considered to be staying at home, and only leading to potential secondary household infections.

### Impact of Releasing All Age Groups

**Figure 4:**
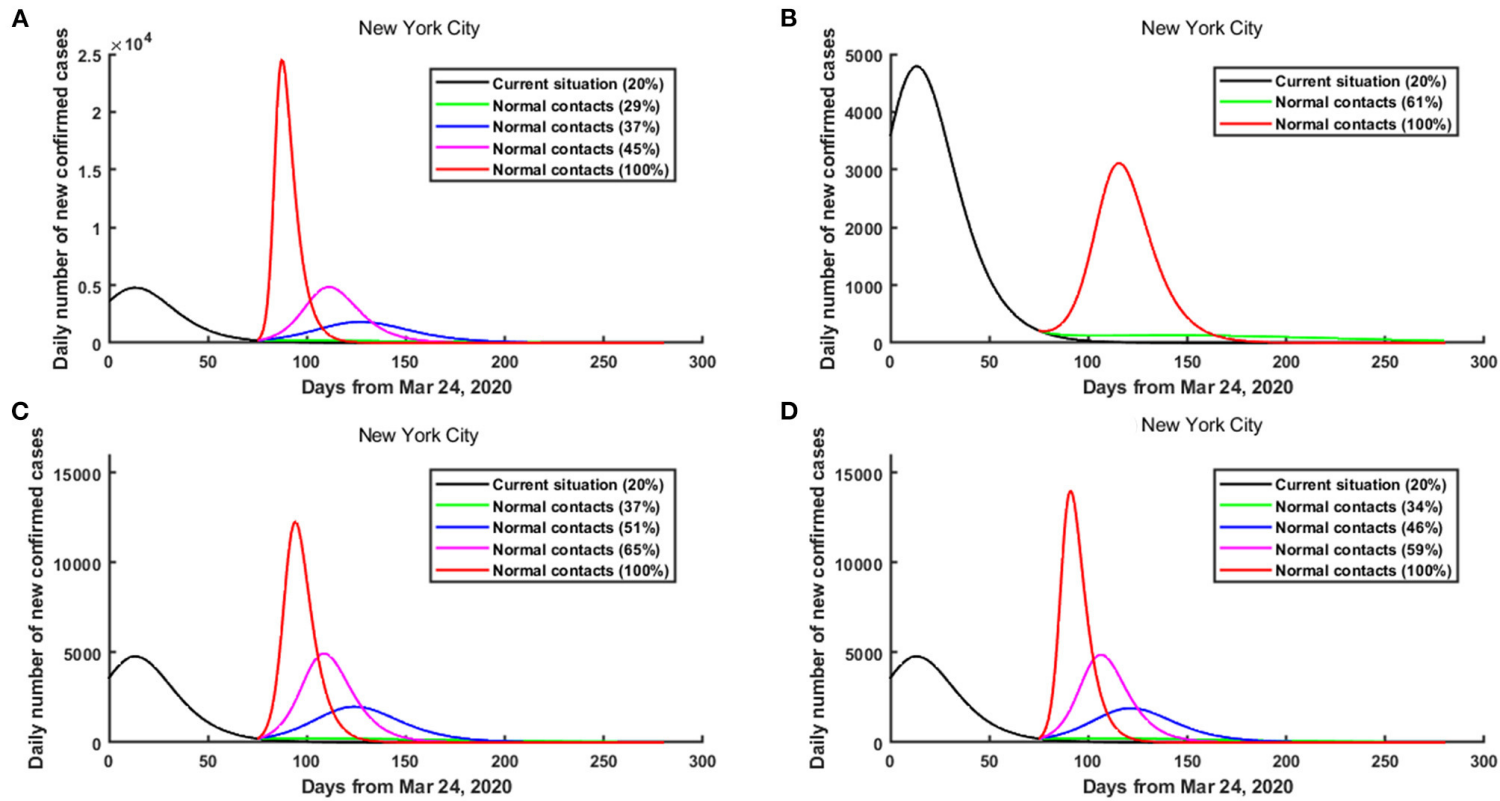
The risk of a second wave of COVID-19 in NYC when different age groups were released from June 8, 2020. **(A)** All age groups were released. **(B)** Only 0–17 age group was released. **(C)** Only 18–44 age group was released. **(D)** Only 45–64 age group was released.

The results showed that under the current practice, the daily number of confirmed cases in NYC reached its peak around April 6, 2020 (with a peak value of 4,787 cases). When all 0–64 age groups in NYC were released from June 8, if the contact rates made by all age groups remained below 29% of the pre-pandemic level, then a second wave of COVID-19 could be prevented in NYC. However, if the contact rates made by all age groups increased to above 45% of the pre-pandemic level, then a second wave could occur, which was greater than the current outbreak (Figure 4A, Supplementary Figure 5). Particularly, if the contact rates made by all age groups increased to 100% of the pre-pandemic level, then the daily number of confirmed cases in NYC reached its peak on June 19, 2020, and the peak value was about 24,490 cases (about 4.12-fold greater than the current outbreak) (Figure 4A, Supplementary Figure 5). As of October 9, compared with the current practice, the cumulative number of confirmed cases in NYC increased by 322,615.

### Impact of Releasing Only One Age Group

The results showed that when only the 0–17 age group in NYC was released from June 8, if the contact rate made by the 0–17 age group remained below 61% of the pre-pandemic level, then a second wave of COVID-19 could be prevented in NYC. Even if the contact rate made by the 0–17 age group increased to 100% of the pre-pandemic level, a greater second wave was unlikely in NYC (Figure 4B, Supplementary Figure 6).

When only the 18–44 age group in NYC was released from June 8, if the contact rate made by the 18–44 age group increased to above 37% of the pre-pandemic level, then a smaller second wave of COVID-19 could occur in NYC (Figure 4C, Supplementary Figure 7). Besides, if the contact rate made by the 18–44 age group increased to above 65% of the pre-pandemic level, then a greater second wave could occur (Figure 4C, Supplementary Figure 7). Particularly, if the contact rate made by the 18–44 age group increased to 100% of the pre-pandemic level, then the daily number of confirmed cases in NYC reached its peak on June 26, 2020, and the peak number was about 12,288 cases (Figure 4C, Supplementary Figure 7). In this case, as of October 9, compared with the current practice, the cumulative number of confirmed cases in NYC increased by 226,966.

When only the 45–64 age group in NYC was released from June 8, if the contact rate made by the 45–64 age group remained below 34% of the pre-pandemic level, then a second wave of COVID-19 could be prevented in NYC (Figure 4D, Supplementary Figure 8). However, if the contact rate made by the 45–64 age group increased to above 59% of the pre-pandemic level, then a second wave could occur, which was greater than the current outbreak (Figure 4D, Supplementary Figure 8).

### Impact of Releasing Two Age Groups

**Figure 5:**
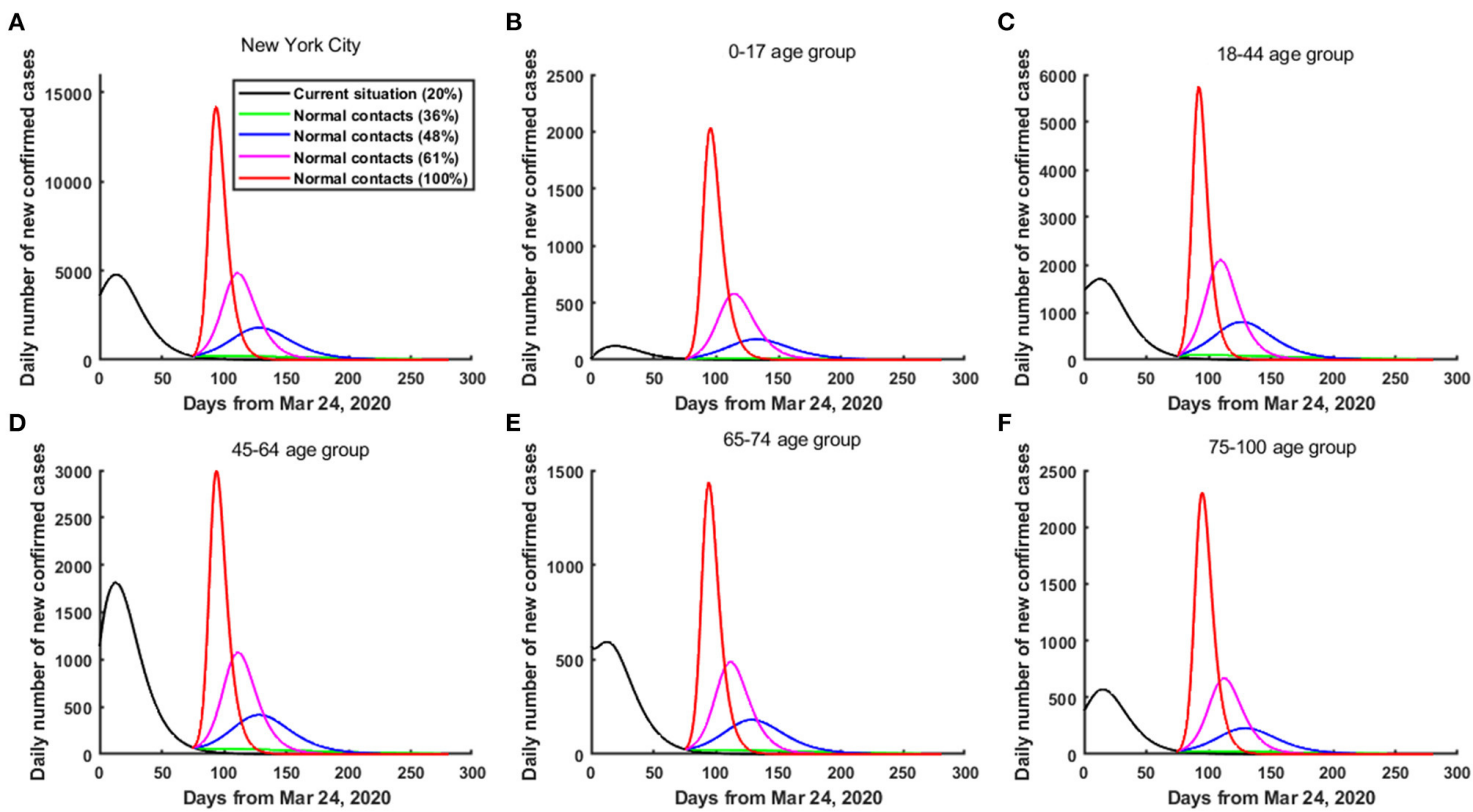
The risk of a second wave of COVID-19 in NYC when only the 0–17 and 18-44 age groups were released from June 8, 2020. **(A)** In the whole population of NYC. **(B)** In the 0–17 age group. **(C)** In the 18–44 age group. **(D)** In the 45–64 age group. **(E)** In the 65–74 age group. **(F)** In the 75–100 age group.

When only the 0–17 and 18–44 age groups in NYC were released from June 8 (Figures 5A–F), if the contact rates made by the 0–17 and 18–44 age groups remained below 36% of the pre-pandemic level, then a second wave of COVID-19 could be prevented in NYC (Figure 5A). However, if the contact rates made by the 0–17 and 18–44 age groups increased to above 61% of the pre-pandemic level, then a greater second wave could occur in NYC (Figure 5A). Particularly, if the contact rates made by the 0–17 and 18–44 age groups increased to 100% of the pre-pandemic level, then the daily number of confirmed cases in NYC reached its peak on June 26, 2020, and the peak value was about 14,203 cases (about 1.97-fold greater than the current outbreak) (Figure 5A). As of October 9, 2020, compared with the current practice, the cumulative number of confirmed cases in NYC increased by 253,443.

**Figure 6:**
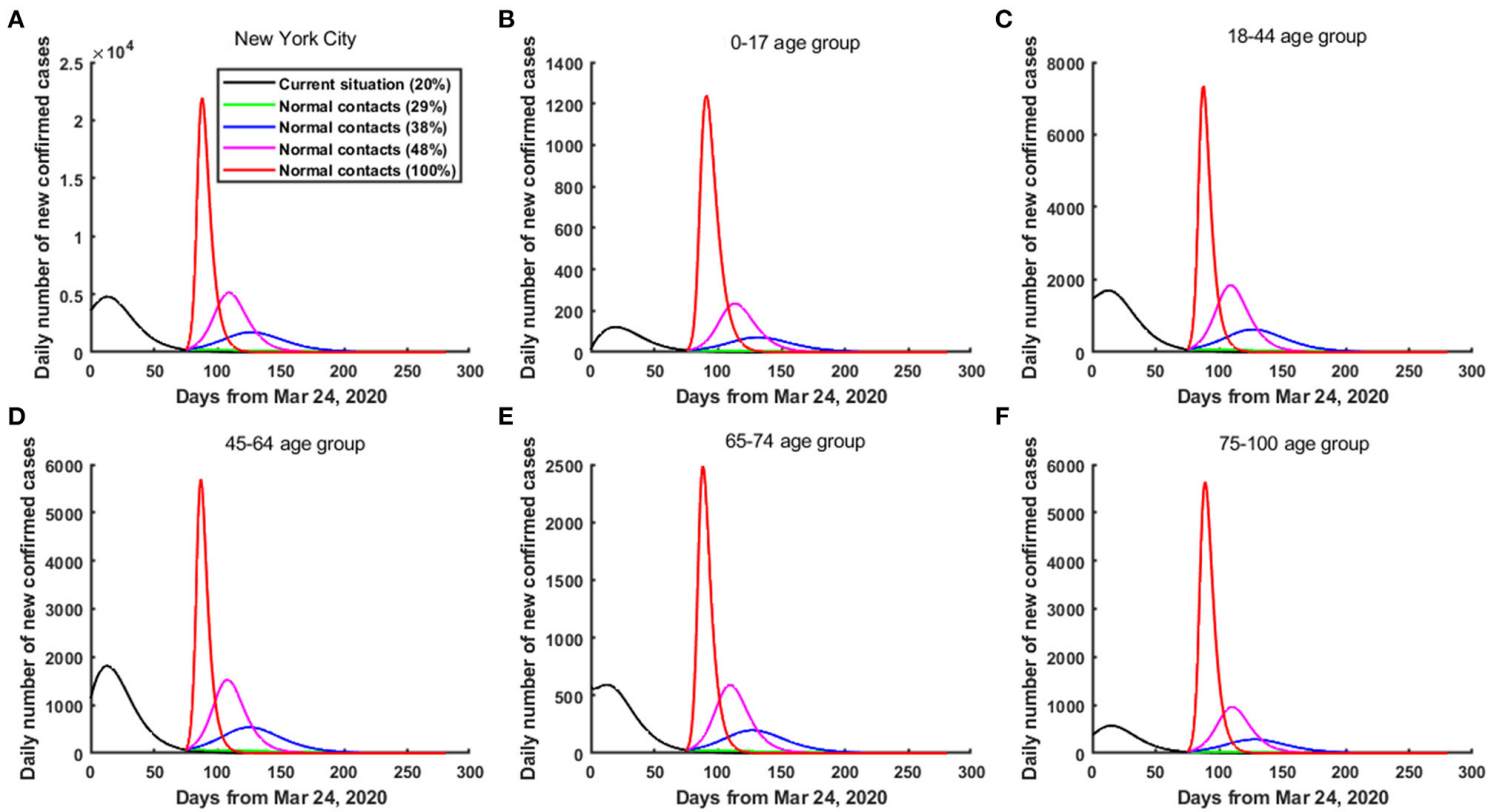
The risk of a second wave of COVID-19 in NYC when only the 18-44 and 45-64 age groups were released from June 8, 2020. **(A)** In the whole population of NYC. **(B)** In the 0–17 age group. **(C)** In the 18–44 age group. **(D)** In the 45–64 age group. **(E)** In the 65–74 age group. **(F)** In the 75–100 age group.

When only the 18–44 and 45–64 age groups in NYC were released from June 8 (Figures 6A–F), if the contact rates made by the 18–44 and 45–64 age groups increased to above 29% of the pre-pandemic level, then a smaller second wave of COVID-19 could occur in NYC (Figure 6A). Furthermore, if the contact rates made by the 18–44 and 45–64 age groups increased to above 48% of the pre-pandemic level, then a greater second wave could occur in NYC (Figure 6A). Particularly, if the contact rates made by the 18–44 and 45–64 age groups increased to 100% of the pre-pandemic level, then the daily number of confirmed cases in NYC reached its peak on June 20, 2020, and the peak value was about 21,933 cases (about 3.58-fold greater than the current outbreak) (Figure 6A). As of October 9, 2020, compared with the current practice, the cumulative number of confirmed cases in NYC increased by 293,094.

### Sensitivity Analysis

The results showed that the cumulative number of deaths in NYC fluctuated within a small range with the different hospitalization rates. Compared with the current practice, if 100, 90, 80, and 70% of all confirmed cases were hospitalized from March 26, 2020, then the cumulative number of deaths in NYC reduced by 11,802, 11,219, 10,543, and 9,758, respectively, and the corresponding percentages reduction were 67.24, 63.9, 60.07, and 55.59%, respectively (Supplementary Figure 9). Besides, if 100, 90, 80, and 70% of all confirmed cases were hospitalized from April 5, 2020, then the percentages reduction in deaths were 63.43, 60.48, 57.01, and 52.93%, respectively (Supplementary Figure 9).

## Discussion

The 2019 novel coronavirus disease (COVID-19) has brought huge challenges to the public health security and economic development in the United States and all over the world. As the epicenter of the COVID-19 pandemic in the United States, how to reduce the risk of COVID-19 transmission more effectively was of great significance to prevent and control COVID-19 in NYC.

In this study, we developed an age-structured compartment model at the population level based on the transmission mechanism of COVID-19 in NYC. In addition, based on three types of reported data for COVID-19 for five age groups (0–17, 18–44, 45–64, 65–74, and 75–100) from March 24, 2020 to June 7, 2020 in NYC, we calibrated the model and estimated the unknown parameters and initial values by using the MCMC approach. Based on the mathematical model and estimated parameters, we evaluated the impact of all diagnosed on the daily number of new infections and the impact of all hospitalized on the cumulative number of deaths, and explored the new relaxing lockdown restriction strategies for different age groups to prevent the second wave in NYC. In particular, we found that hospitalizing all cases led to better control of the epidemic and reduced the mortality. On the one hand, the mortality was correlated with health-care burden, and the death rate of hospitalized cases decreased over time as more healthcare resources became available (28, 29). In fact, the adequate healthcare resources helped to improve the treatment conditions and reduced the mortality of severe COVID-19 outside Hubei Province (28, 29, 33). On the other hand, the adequate mobile cabin hospitals conducted centralized isolation and scientific treatment early for confirmed cases with mild symptoms, which also helped to better relieve patients' conditions and reduce the risk of severe illness. Furthermore, to centralized isolation and treatment these confirmed cases with mild symptoms prevented them from further spreading the disease as a source of infection. Our results may provide a quantitative reference for policy-making to further prevent and control COVID-19 in NYC.

The innovations of this study were reflected in the following four aspects. First, we considered the age differences among different populations, more importantly, we estimated the age-specific parameters such as the contact rates and the death rates of hospitalized cases based on actual reported data. Compared with the other existing mathematical models (10, 22, 34), our estimated parameters and predicted results were more in line with the actual situation of NYC and could better help to make the releasing strategy. Second, we calibrated the model and estimated the unknown parameters based on three types of reported data for COVID-19 in NYC, including the cumulative number of confirmed cases, the cumulative number of deaths and the cumulative number of hospitalizations for five age groups (0–17, 18–44, 45–64, 65–74, and 75–100). Compared with using only one type of reported data or two types of reported data to estimate the unknown parameters, using three types of reported data to estimate the unknown parameters reduced the error and uncertainty of the parameter estimation, and the estimated parameters were more reasonable and reliable. Third, we evaluated the impact of all diagnosed and all hospitalized on the development trend of COVID-19 in NYC. If there were enough mobile cabin hospitals in NYC, just like in Wuhan, China, then the cumulative number of deaths could be significantly reduced in NYC. Finally, based on the development trend of COVID-19 in NYC, we explored the new relaxing lockdown restriction strategies from an age perspective.

There were also some limitations in this study. First, we assumed that the contact matrix was symmetric. Second, we ignored the death rates of freely infected individuals and confirmed cases who stayed at home. Third, we ignored the heterogeneity of the population and assumed the whole population was homogeneously distributed. Fourth, due to the limitation of reported data, we did not distinguish the latent population and asymptomatic infected population. Besides, we did not consider the effect of re-infection for the recovered cases as it was not easy to know how many people would occur re-infection due to the limitation of current research and reported reinfected data. Moreover, we did not explicitly use specific parameters to represent other non-pharmacological interventions, such as face masks and social distancing, although they were implicitly integrated into the transmission rate and the contact rate. Finally, the effect of vaccination on the model was not considered since during the time of this study, the vaccine was unavailable.

In conclusion, in this study, we found that the earlier the hospitalization of all confirmed cases, the greater the reduction in the total number of deaths. The earlier the diagnosis of all infected people, the greater the reduction in the daily number of new infected individuals in NYC. Therefore, if NYC referred to the experience from the Wuhan mobile cabin hospital in controlling the COVID-19, then a second wave could be avoided. In addition, maintaining social distancing still played an important role in preventing the resurgence of the epidemic in NYC.

## Data Availability Statement

The original contributions presented in the study are included in the article/Supplementary Material, further inquiries can be directed to the corresponding authors.

## Author Contributions

JZ and FJ participated in the design of the manuscript. ML and JZ analyzed the data and drafted the manuscript. ML, ZL, MS, YL, and FJ analyzed the data and participated the discussion on the manuscript. JZ, YL, and FJ revised the manuscript. All authors have read and approved the final version of the manuscript.

## Conflict of Interest

The authors declare that the research was conducted in the absence of any commercial or financial relationships that could be construed as a potential conflict of interest.

## Publisher's Note

All claims expressed in this article are solely those of the authors and do not necessarily represent those of their affiliated organizations, or those of the publisher, the editors and the reviewers. Any product that may be evaluated in this article, or claim that may be made by its manufacturer, is not guaranteed or endorsed by the publisher.
