# Supplementary material for: How to Reduce the Transmission Risk of COVID-19 More Effectively in New York City: An Age-Structured Model Study
Source: Front Med (Lausanne). 2021 Aug 13;8:641205. doi: 10.3389/fmed.2021.641205 (PMC8414980; doi:10.3389/fmed.2021.641205)
Supplement: Supplementary file 2 [file Data_Sheet_1.docx]

**Supplementary Material**

**How to reduce the transmission risk of COVID-19 more effectively in New York City: an age-structured model study**

Miaolei Li^1^, Jian Zu^1,*^, Zongfang Li^2,3^, Mingwang Shen^4^, Yan Li^5,6^, Fanpu Ji^2,3,7,*^

1 School of Mathematics and Statistics, Xi’an Jiaotong University, Xi’an 710049, China; [jianzu@xjtu.edu.cn (J.Z.)](mailto:jianzu@xjtu.edu.cn%20(J.Z.)); [2454816940@qq.com](mailto:2454816940@qq.com) (M.L.L.);

2 National & Local Joint Engineering Research Center of Biodiagnosis and Biotherapy, The Second Affiliated Hospital of Xi’an Jiaotong University, Xi’an 710004, China; [lzf2568@xjtu.edu.cn](mailto:lzf2568@xjtu.edu.cn)

3 Key Laboratory of Environment and Genes Related to Diseases, Xi'an Jiaotong University, Ministry of Education of China, Xi'an 710061, China

4 School of Public Health, Health Science Center, Xi’an Jiaotong University, Xi’an 710061, China; [mingwangshen521@xjtu.edu.cn](mailto:mingwangshen521@xjtu.edu.cn)

5 Department of Population Health Science and Policy, Icahn School of Medicine at Mount Sinai, New York, NY, USA;

6 Department of Obstetrics, Gynecology, and Reproductive Science, Icahn School of Medicine at Mount Sinai, New York, NY, USA; yan.li1@mountsinai.org.

7 Department of Infectious Disease,s The Second Affiliated Hospital of Xi’an Jiaotong University, Xi’an 710004, China; [jifanpu1979@163.com](mailto:jifanpu1979@163.com) or [infection@xjtu.edu.cn](mailto:infection@xjtu.edu.cn)

***Correspondence: Jian Zu**, School of Mathematics and Statistics, Xi’an Jiaotong University, Xi’an, Shaanxi, 710049, P. R. China (Email address: [jianzu@xjtu.edu.cn](mailto:jianzu@xjtu.edu.cn)).

**Fanpu Ji**, Department of Infectious Diseases, the Second Affiliated Hospital of Xi’an Jiaotong University, 157 Xi Wu Road, Xi'an 710004, Shaanxi Province, PR. China. (Email address:[jifanpu1979@163.com](mailto:jifanpu1979@163.com) or infection@xjtu.edu.cn).

This is a supplementary material describing in detail the formulation of transmission model and parameter estimation presented in the main text.

1. **Model formulation**

Based on the natural history and the transmission mechanism of COVID-19 in New York City, we developed an age-structured susceptible-infected-confirmed-hospitalized-recovered (SICHR) compartment model at the population level by considering two different quarantined routes [1-9]. The confirmed cases with mild symptoms were quarantined at home, and the confirmed cases with severe symptoms were quarantined and treated at hospital. Specifically, we assumed that the maximum age of people was 100 years old and divided the total population of New York City into 5 age groups: 0-17, 18-44, 45-64, 65-74 and 75-100 years [10]. The population in New York City in each age group $i$ were divided into five compartments: Susceptible individuals$S_{i}(t)$; Infected individuals$I_{i}(t)$; Confirmed cases$C_{i}(t)$; Hospitalized cases$H_{i}(t)$ and Recovered cases$R_{i}(t)$.

For simplicity, we made the following assumptions:

1. The whole population in New York City was homogeneously distributed.
2. The susceptibility for the whole susceptible individuals was assumed to be the same.
3. The contact matrix was symmetric.
4. The death rates of freely infected individuals and confirmed cases who stayed at home were not considered.
5. The recovered cases would not be infected again.

Based on the above assumptions and **Figure 1** (in the main text), the age-structured SICHR compartment model at the population level was described by equations (1) and (2).

$\begin{matrix} dS_{i}/dt=-\lambda(t)S_{i}, \\ dI_{i}/dt=\lambda(t)S_{i}-\frac{\delta_{i}}{\rho}I_{i}-\gamma_{0}I_{i}, \\ dC_{i}/dt=\frac{\delta_{i}}{\rho}I_{i}-\frac{\alpha_{i}}{k}C_{i}-\gamma_{1}C_{i}, \\ dH_{i}/dt=\frac{\alpha_{i}}{k}C_{i}-\mu_{i}H_{i}-\gamma_{2}H_{i}, \\ dR_{i}/dt=\gamma_{0}I_{i}+\gamma_{1}C_{i}+\gamma_{2}H_{i}. \end{matrix}$ (1)

where $\lambda(t)=\beta\sum_{j=1}^{5} c_{ij}\frac{(I_{j}(t)+\theta C_{j}(t))}{N_{j}(t)}$,$S(t)=\sum_{i=1}^{5} S_{i}(t)$, $I(t)=\sum_{i=1}^{5} I_{i}(t)$, $C(t)=\sum_{i=1}^{5} C_{i}(t)$, $R(t)=\sum_{i=1}^{5} R_{i}(t)$, $N(t)=S(t)+I(t)+R(t)$. The description of parameters and variables as well as their corresponding 95% confidence intervals were summarized in **Table S1**.

The cumulative number of confirmed cases $Y_{i}$ in age group$i$, the cumulative number of deaths $Z_{i}$ in age group $i$ and the cumulative number of hospitalizations$P_{i}$ in age group $i$ were given by:

$\begin{matrix} dY_{i}/dt=\frac{\delta_{i}}{\rho}I_{i}(t), \\ dZ_{i}/dt=\mu_{i}H_{i}(t), \\ dP_{i}/dt=\frac{\alpha_{i}}{k}C_{i}(t). \end{matrix}$ (2)

1. **Initial conditions of model (1) and (2) and input parameters**

We chose March 24, 2020 as the initial time of models (1) and (2) because March 24, 2020 was the initial time of the reported data we collected. In addition, New York City had implemented stay-at-home orders from March 23, 2020. Therefore, the frequency of public contact rates had resulted in a significant reduction in the whole population since March 24, 2020 in New York City [11].

The initial values of recovered individuals $R_{i}(0)$were assumed to be 0 because there was no recovered case in New York City as of March 24, 2020. The initial values of confirmed cases $C_{i}(0)$ were calculated approximately based on the equation:$C_{i}(0)=Y_{i}(0)-P_{i}(0)-R_{i}(0)$.The initial values of the existing hospitalizations $H_{i}(0)$ were calculated approximately based on the equation: $H_{i}(0)=P_{i}(0)-R_{i}(0)$. The total population in New York City was 8,398,748 based on U.S. Census Bureau-American Community Survey in 2018 [12]. According to the age-specific proportion of the population in New York City in 2017 [13], we calculated the total population $N_{i}(0)$ in each age group $i$ in New York City. The initial values of the existing susceptible individuals $S_{i}(0)$ were calculated approximately based on the equation:$S_{i}(0)=N_{i}(0)-I_{i}(0)-C_{i}(0)-H_{i}(0)-R_{i}(0)$. The initial values of the existing infected individuals $I_{i}(0)$ and other unknown parameters in the model were estimated with the reported data. By using the Markov Chain Monte Carlo (MCMC) method and setting the iteration number to 8,000 and the first 7,500 times as burn-in periods, we obtained the estimated initial values and unknown parameters in model (1) as well as the corresponding 95% confidence intervals. The description of parameters and variables as well as their corresponding 95% confidence intervals were summarized in **Table S1**.

1. **Supplementary Tables**

**Table S1. Parameter description for models (1) and (2)**

| Parameters | Meanings | Values | 95% CI | References |
| --- | --- | --- | --- | --- |
| $\beta$ | Per-capita transmission rate | 0.0325 | (0.0324, 0.0325) | MCMC |
| $c_{11}$ | Contact rate of the age group 0-17 years made  by age group 0-17 years | 2.1611 | (2.1611, 2.1611) | MCMC |
| $c_{12}$ | Contact rate of the age group 18-44 years made  by age group 0-17 years | 0.7851 | (0.7851, 0.7852) | MCMC |
| $c_{13}$ | Contact rate of the age group 45-64 years made  by age group 0-17 years | 0.2634 | (0.2634, 0.2635) | MCMC |
| $c_{14}$ | Contact rate of the age group 65-74 years made  by age group 0-17 years | 1.3731e-04 | (0.1300e-03, 0.1446e-03) | MCMC |
| $c_{15}$ | Contact rate of the age group 75-100 years made by age group 0-17 years | 2.4072e-05 | (0.0772e-04, 0.4042e-04) | MCMC |
| $c_{22}$ | Contact rate of the age group 18-44 years made by age group 18-44 years | 3.3636 | (3.3636, 3.3636) | MCMC |
| $c_{23}$ | Contact rate of the age group 45-64 years made by age group 18-44 years | 2.1656 | (2.1655, 2.1656) | MCMC |
| $c_{24}$ | Contact rate of the age group 65-74 years made by age group 18-44 years | 2.0419 | (2.0419, 2.0420) | MCMC |
| $c_{25}$ | Contact rate of the age group 75-100 years made by age group 18-44 years | 0.5025 | (0.5025, 0.5026) | MCMC |
| $c_{33}$ | Contact rate of the age group 45-64 years made by age group 45-64 years | 4.7424 | (4.7423, 4.7424) | MCMC |
| $c_{34}$ | Contact rate of the age group 65-74 years made by age group 45-64 years | 2.8968 | (2.8968, 2.8968) | MCMC |
| $c_{35}$ | Contact rate of the age group 75-100 years made by age group 45-64 years | 1.0706 | (1.0705, 1.0706) | MCMC |
| $c_{44}$ | Contact rate of the age group 65-74 years made by age group 65-74 years | 1.9445 | (1.9445, 1.9445) | MCMC |
| $c_{45}$ | Contact rate of the age group 75-100 years made by age group 65-74 years | 0.2476 | (0.2476, 0.2477) | MCMC |
| $c_{55}$ | Contact rate of the age group 75-100 years made by age group 75-100 years | 3.0835e-05 | (0.2279e-04, 0.3888e-04) | MCMC |
| $\delta_{1}$ | Transfer rate from free infected individuals to confirmed cases in the age group 0-17 years | 0.0337 | (0.0337, 0.0337) | MCMC |
| $\delta_{2}$ | Transfer rate from free infected individuals to confirmed cases in the age group 18-44 years | 0.0532 | (0.0532, 0.0533) | MCMC |
| $\delta_{3}$ | Transfer rate from free infected individuals to confirmed cases in the age group 45-64 years | 0.0745 | (0.0744, 0.0746) | MCMC |
| $\delta_{4}$ | Transfer rate from free infected individuals to confirmed cases in the age group 65-74 years | 0.1060 | (0.1058, 0.1061) | MCMC |
| $\delta_{5}$ | Transfer rate from free infected individuals to confirmed cases in the age group 75-100 years | 0.5763 | (0.5760, 0.5766) | MCMC |
| $\alpha_{1}$ | Transfer rate from confirmed cases to hospitalized cases in the age group 0-17 years | 0.0535 | (0.0533, 0.0537) | MCMC |
| $\alpha_{2}$ | Transfer rate from confirmed cases to hospitalized cases in the age group 18-44 years | 0.0661 | (0.0660, 0.0662) | MCMC |
| $\alpha_{3}$ | Transfer rate from confirmed cases to hospitalized cases in the age group 45-64 years | 0.1869 | (0.1868, 0.1870) | MCMC |
| $\alpha_{4}$ | Transfer rate from confirmed cases to hospitalized cases in the age group 65-74 years | 0.5140 | (0.5139, 0.5141) | MCMC |
| $\alpha_{5}$ | Transfer rate from confirmed cases to hospitalized cases in the age group 75-100 years | 0.9527 | (0.9526, 0.9529) | MCMC |
| $k$ | Average time from confirmed cases to hospitalized cases | 3.1973 | (3.1972, 3.1974) | MCMC |
| $\mu_{1}$ | Death rate of hospitalized cases in the age group 0-17 years | 0.0032 | (0.0032, 0.0032) | MCMC |
| $\mu_{2}$ | Death rate of hospitalized cases in the age group 18-44 years | 0.0127 | (0.0126, 0.0128) | MCMC |
| $\mu_{3}$ | Death rate of hospitalized cases in the age group 45-64 years | 0.0353 | (0.0352, 0.0355) | MCMC |
| $\mu_{4}$ | Death rate of hospitalized cases in the age group 65-74 years | 0.0731 | (0.0731, 0.0732) | MCMC |
| $\mu_{5}$ | Death rate of hospitalized cases in the age group 75-100 years | 0.1636 | (0.1635, 0.1636) | MCMC |
| $\gamma_{0}$ | Recovery rate of infected individuals in the free environment | 0.2091 | (0.2088, 0.2094) | MCMC |
| $\gamma_{1}$ | Recovery rate of confirmed cases | 0.1791 | (0.1785, 0.1797) | MCMC |
| $\gamma_{2}$ | Recovery rate of hospitalized cases | 0.1279 | (0.1279, 0.1280) | MCMC |
| $\theta$ | Relative transmission strength of confirmed cases to the infected individuals in the free environment | 0.0111 | (0.0110, 0.0112) | MCMC |
| $\rho$ | Average time from infected individuals in the free environment to confirmed cases | 4.9985 | (4.9974, 4.9997) | MCMC |
| $I_{1}(0)$ | Initial value of free infected individuals in the age group 0-17 years | 2.0305e+03 | (1.9981e+03, 2.0629e+03) | MCMC |
| $I_{2}(0)$ | Initial value of free infected individuals in the age group 18-44 years | 1.3872e+05 | (1.3858e+05, 1.3885e+05) | MCMC |
| $I_{3}(0)$ | Initial value of free infected individuals in the age group 45-64 years | 7.6096e+04 | (7.6052e+04, 7.6140e+04) | MCMC |
| $I_{4}(0)$ | Initial value of free infected individuals in the age group 65-74 years | 2.6973e+04 | (2.6929e+04, 2.7016e+04) | MCMC |
| $I_{5}(0)$ | Initial value of free infected individuals in the age group 75-100 years | 3.3155e+03 | (3.2674e+03, 3.3635e+03) | MCMC |

1. **Supplementary Figures**


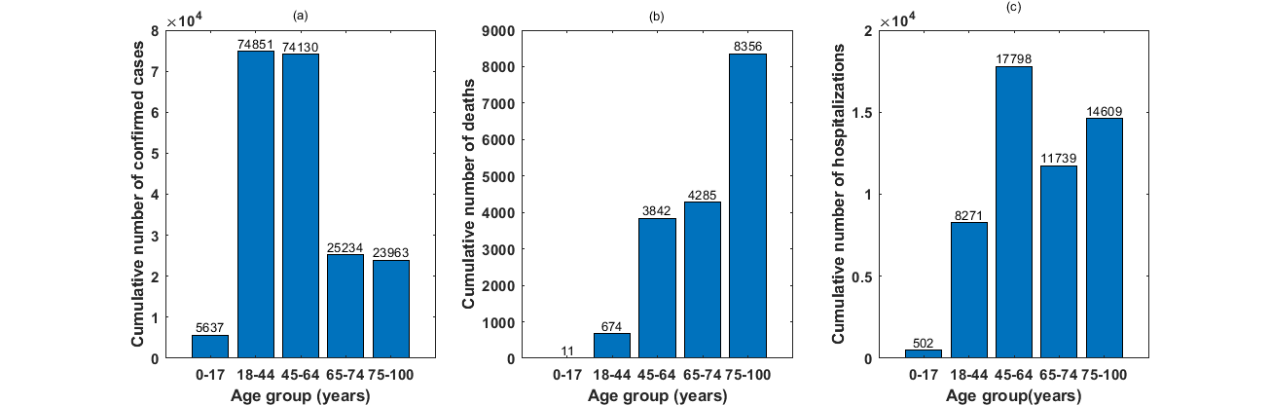


**Figure S1. The reported data for COVID-19 in New York City as of June 7, 2020.** (a) Cumulative number of confirmed cases in each age group as of June 7, 2020. (b) Cumulative number of deaths in each age group as of June 7, 2020. (c) Cumulative number of hospitalizations in each age group as of June 7, 2020.

**Figure S2. Comparison of estimated cumulative number of confirmed cases with the observed data.** (a) In the whole population of New York City. (b) In the 0-17 age group. (c) In the 18-44 age group. (d) In the 45-64 age group. (e) In the 65-74 age group. (f) In the 75-100 age group.

**Figure S3. Comparison of estimated cumulative number of deaths with the observed data.** (a) In the whole population of New York City. (b) In the 0-17 age group. (c) In the 18-44 age group. (d) In the 45-64 age group. (e) In the 65-74 age group. (f) In the 75-100 age group.

**Figure S4. Comparison of estimated cumulative number of hospitalizations with the observed data.** (a) In the whole population of New York City. (b) In the 0-17 age group. (c) In the 18-44 age group. (d) In the 45-64 age group. (e) In the 65-74 age group. (f) In the 75-100 age group.

**Figure S5. The risk of a second wave of COVID-19 in New York City when 0-64 age groups were released from June 8, 2020.** (a) In the whole population of New York City. (b) In the 0-17 age group. (c) In the 18-44 age group. (d) In the 45-64 age group. (e) In the 65-74 age group. (f) In the 75-100 age group.

**Figure S6. The risk of a second wave of COVID-19 in New York City when only 0-17 age group was released from June 8, 2020.** (a) In the whole population of New York City. (b) In the 0-17 age group. (c) In the 18-44 age group. (d) In the 45-64 age group. (e) In the 65-74 age group. (f) In the 75-100 age group.

**Figure S7. The risk of a second wave of COVID-19 in New York City when only 18-44 age group was released from June 8, 2020.** (a) In the whole population of New York City. (b) In the 0-17 age group. (c) In the 18-44 age group. (d) In the 45-64 age group. (e) In the 65-74 age group. (f) In the 75-100 age group.

**Figure S8. The risk of a second wave of COVID-19 in New York City when only 45-64 age group was released from June 8, 2020.** (a) In the whole population of New York City. (b) In the 0-17 age group. (c) In the 18-44 age group. (d) In the 45-64 age group. (e) In the 65-74 age group. (f) In the 75-100 age group.

**Figure S9. Impact of different hospitalization rates on the cumulative number of deaths in New York City.** (a) The effect of all hospitalized on the cumulative number of deaths in New York City. (b) The effect of 90% hospitalized on the cumulative number of deaths in New York City. (c) The effect of 80% hospitalized on the cumulative number of deaths in New York City. (d) The effect of 70% hospitalized on the cumulative number of deaths in New York City.

**References**

1. Kretzschmar ME, Rozhnova G, Bootsma MCJ, van Boven M, van de Wijgert JHHM, Bonten MJM. Impact of delays on effectiveness of contact tracing strategies for COVID-19: a modelling study. Lancet Public Health, 2020, 5(8):e452-e459.
2. Grassly NC, Pons-Salort M, Parker EPK, White PJ, Ferguson NM; Imperial College COVID-19 Response Team. Comparison of molecular testing strategies for COVID-19 control: a mathematical modelling study. Lancet Infect Diseases, 2020, 20(12):1381-1389.
3. Hao X, Cheng S, Wu D, Wu T, Lin X, Wang C. Reconstruction of the full transmission dynamics of COVID-19 in Wuhan. Nature, 2020, 584(7821):420-424.
4. Giordano G, Blanchini F, Bruno R, et al. Modelling the COVID-19 epidemic and implementation of population-wide interventions in Italy. Nature Medicine, 2020, 26(6):855-860.
5. Prem K, Liu Y, Russell TW, et al. The effect of control strategies to reduce social mixing on outcomes of the COVID-19 epidemic in Wuhan, China: a modelling study. Lancet Public Health, 2020, 5(5):e261-e270.
6. Flaxman S, Mishra S, Gandy A, et al. Estimating the effects of non-pharmaceutical interventions on COVID-19 in Europe. Nature, 2020, 584(7820): 257-261.
7. Worby CJ, Chang HH. Face mask use in the general population and optimal resource allocation during the COVID-19 pandemic. Nature Communications, 2020, 11(1): 4049.
8. Ullah S, Khan MA. Modeling the impact of non-pharmaceutical interventions on the dynamics of novel coronavirus with optimal control analysis with a case study. Chaos, Solitons & Fractals, 2020, 139:110075.
9. Alqarni MS, Alghamdi M, Muhammad T, Alshomrani AS, Khan MA. Mathematical modeling for novel coronavirus (COVID-19) and control. Numerical Methods for Partial Differential Equation, 2020, doi:10.1002/num.22695.
10. NYC Coronavirus Disease 2019 (COVID-19) Data. <https://github.com/nychealth/coronavirus-data.>
11. Gu J, Li J, Hao Y, et al. Bulletin of COVID-19 online survey. http://sph.sysu.edu.cn/article/1257 [Accessed 4 Mar 2020].
12. [NYCdata: Metropolitan Statistical Area (MSA) Population](https://www.baruch.cuny.edu/nycdata/population-geography/population.htm). <https://www.baruch.cuny.edu/nycdata/population-geography/population.htm.>
13. New York City (NYC) Age and Sex Distribution-By County. <https://www.baruch.cuny.edu/nycdata/population-geography/age_distribution.htm.>
